# Photoacoustic effect applied on model membranes and living cells: direct observation with multiphoton excitation microscopy and long-term viability analysis

**DOI:** 10.1038/s41598-019-56799-9

**Published:** 2020-01-15

**Authors:** Francisco Galisteo-González, Bingen G. Monasterio, David Gil, Mikel Valle, Félix M. Goñi

**Affiliations:** 1 Departamento de Física Aplicada, Universidad de Granada, 18570 Granada, Spain; 2 Instituto Biofisika (UPV/EHU, CSIC), Barrio Sarriena s/n, 48940 Leioa, Spain; 3 Departamento de Bioquímica y Biología Molecular, Universidad del País Vasco, Barrio Sarriena s/n, 48940 Leioa, Spain; 4 Structural Biology Unit, Center for Cooperative Research in Biosciences, CIC bioGUNE, Derio, Spain

**Keywords:** Membrane biophysics, Permeation and transport

## Abstract

The photoacoustic effect is generated when a variable light interacts with a strongly light-absorbing material. In water, it may produce hot bubbles and shock waves that could affect the integrity of nearby cellular membranes, opening transient pores (photoporation). In this study, we have evaluated the effect of pulsed laser-irradiated carbon nanoparticles (cNP) on model membranes and on Chinese hamster ovary (CHO) cells. Fluorescence lifetime measurements of calcein-loaded liposomes support the notion that the photoacoustic effect causes transient openings in membranes, allowing diffusion fluxes driven by gradient concentrations. With CHO cells, we have shown that this effect can induce either intracellular delivery of calcein, or release of cellular compounds. The latter process has been recorded live with multiphoton excitation microscopy during pulsed infrared laser irradiation. Calcein loading and cell viability were assayed by flow cytometry, measuring necrotic cells as well as those in early apoptosis. To further assess long-term cell recovery after the rather harsh treatment, cells were reseeded and their behaviour recorded for 48 h. These extended studies on cell viability show that pulsed laser cNP photoporation may be considered an adequate intracellular delivery technique only if employed with soft irradiation conditions (below 50 mJ/cm^2^).

## Introduction

Biological membranes constitute the cell boundaries, isolating the inner cell medium from the external environment. They consist of a hydrophobic matrix, formed by an oriented double layer of phospholipids (and/or glycolipids) to which proteins are bound in different forms^1^. This barrier prevents large molecules from entering cells in a passive way. Only by active mechanisms, like receptor-mediated endocytosis, can they reach the cytoplasm^2^. This is the basis of specific or receptor-targeted nanoparticle technology^3,4^. However, this process may be slow, and it often leads molecules ultimately into lysosomes, thus to enzymatic degradation^5^. In order to promote the introduction of drugs or genetic material avoiding the endocytic pathway, methods for direct cytoplasmic delivery are necessary^6^.

Different physical forces have been employed to induce transient openings in cell membranes, which can close fast enough as to not jeopardize cell living conditions. Examples of such methods are microinjection^7^, ultrasound^8^, electroporation^9^ or light- irradiation techniques^10^. In the latter category, the so-called photoacoustic effect is generated when a variable light interacts with a strong light-absorbent material *e.g*. black carbon^11,12^. In aqueous media this effect can be easily achieved by irradiating a carbon nanoparticle (cNP) suspension with a pulsed infrared (IR) laser. Light energy absorption heats cNP above 1000 °C^13,14^. Vaporization of the surrounding water and generation of acoustic emissions can impact nearby cells thermally -by contact with the hot bubbles- and mechanically -by fluid mechanical forces- to physically disrupting cell membranes with transient holes, as proposed in the seminal works in this field of Dr. Prausnitz group^13–19^. They have called this phenomenon “transient nanoparticle energy transduction” (TNET).

This kind of cNP did not cause cytotoxicity in human prostate adenocarcinoma cells DU145 at concentrations lower than 200 mg/L^15^, five times higher than the maximum concentration used in the experiments here reported. Additional examples of photoacoustic spectroscopy as applied to molecular imaging are known^20–23^, see review by Chen *et al*.^23^.With other forms of carbon nanosystems, *e.g*. graphene or carbon nanotubes, controversy exists about their possible toxicity in biomedical applications^24–26^.

To corroborate the occurrence of these transient membrane holes, we have investigated the effect of the photoacoustic treatment on liposomes, as model cell membranes, to gain insight into the mechanism by which membranes are disrupted by this kind of physical distress. To this aim vesicles were loaded with fluorescent water-soluble molecules, and vesicle leakage was studied by measuring fluorescence lifetimes of the corresponding dye in vesicle suspensions prepared and treated in different ways. In the same way, intracellular delivery to and release from Chinese hamster ovarian (CHO) cells were monitored by confocal and multiphoton excitation microscopy.

Multiphoton (or Two-Photon) Excitation Microscopy is an advanced imaging technique based on the principle that two photons of red or infrared wavelength reaching a fluorophore at the same time cause the same excitation as a photon with twice the frequency, but avoiding the problems concerning high energy radiation^27,28^. The confocal microscope is equipped with an ultra-fast (femtosecond) pulsed laser tuneable in the IR region, that can irradiate the sample simultaneously with the lasers for conventional fluorescence imaging. We have used the advanced technology of this device with a novel and different purpose: to investigate the photoacoustic effect acting on living cells. If a cell suspension is exposed to pulsed IR light in the presence of cNP, and is simultaneously irradiated with another laser light, the fate of a fluorophore can be monitored in real time. This method has been used to monitor the liberation of a substance from a living cell promoted by the photoacoustic effect.

However, this method could cause cell damage and compromise viability. Such damage may not be immediately detectable. It is then advisable to study cell viability not only in the short-term, but also over a more extended period of time, trying to elucidate if cells retain their basic functions intact after irradiation. Previous studies describing this effect analysed the influence of laser-activated cNP on cell viability by counting only necrotic cells (propidium iodide stained) in a flow cytometer. We have extended the analysis by including also those in early apoptosis, and studying cell viability by re-seeding of the cells on flat flasks. Long-term cell survival can only be expected if they are able, after the laser treatment, to adhere again to a surface, spread and divide trying to cover the surface (confluence). We propose that these long-term studies on cell viability are necessary to support the idea that the photoacoustic effect is a valid intracellular delivery technique for living cells.

## Results and Discussion

### Nanoparticle characterization

Size distribution of the cNP suspension was determined by DLS. The particles displayed an average diameter of 204 ± 4 nm (intensity-weighted Z-average ± standard deviation, n = 3 different preparations) and an average polydispersity index (PDI) of 0.08 ± 0.02. This low value indicates a good monodispersity (homogeneity) of the suspension.

**Figure 1:**
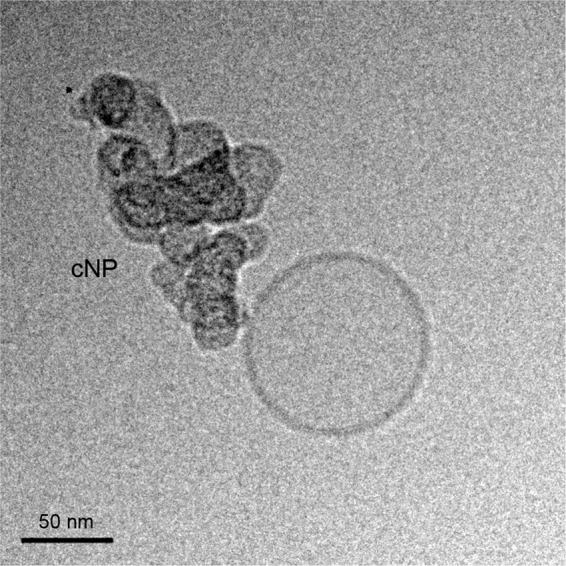
Representative cryo-TEM image of a cNP and a liposome. Image captured using DigitalMicrograph software (version 3.31.2360.0; https://www.gatan.com).

Figure 1 shows a cryo-TEM image of a representative cNP. The Supplementary Fig. S1 shows a small image gallery of vesicles with and without cNP. The nanoparticles do not present a regular shape, and seem to be composed of aggregates of small units of *ca*. 20–30 nm, bound together giving rise to irregular structures. We tried to break down these aggregates with high energy probe-sonication, but size distribution did not vary significantly, revealing that these structures are quite stable, at least respect to mechanical forces.

### Photoacoustic effect on model membranes

Our first goal was to get some insight into the possible cell disruption mechanisms studying vesicle leakage through fluorescent lifetime measurements. To this aim, we loaded large unilamellar vesicles (LUV) with highly concentrated fluorophore calcein (70 mM) and removed the non-entrapped fluorophore by size exclusion chromatography. The entrapped dye displays little fluorescence due to self-quenching, and its fluorescent emission increases greatly when it is diluted upon release from the vesicles. However, this fluorescence increase does not allow distinguishing whether a partial dye release (efflux) comes from a fraction of the vesicles being completely emptied while others remain intact (all-or-none mechanism, caused by slow-closing or permanent holes), or from most/all the vesicles releasing only a part of their content and keeping the rest (graded mechanism, only compatible with fast-closing transient openings). These mechanisms, nevertheless, can be distinguished by fluorescence lifetime measurements^29^, since entrapped self-quenched calcein displays a lifetime τ_E_ = 0.4 ns, smaller than the value measured for diluted (5 μM) free calcein, τ_F_ = 4.0 ns.

To assess the effectiveness of the procedure, different mixtures of calcein-entrapped vesicles and free calcein (ranging from 0 up to 100% of efflux and simulating a continuous process of all-or-none vesicle leakage) were prepared and analysed by measuring fluorescence lifetimes. Various theoretical approaches were applied to the parameter values derived from the experimental curve biexponential fitting, namely *B*_1,_ *B*_2_, τ_1_ and τ_2_, which can be associated with free (1) and entrapped (2) values, respectively^29^. The best results to estimate efflux were obtained by using only the first (*B*_1_) parameter, just the one related with free calcein, in the form:

1 $$E\,( \% )=\frac{{B}_{1}-{B}_{0}}{{B}_{100}-{B}_{0}}\cdot 100$$

where *B*_0_ is the *B*_1_ value obtained for a freshly prepared vesicle solution, while *B*_100_ is the same value for a diluted calcein solution at the same concentration (as determined by disruption of the previous sample with Triton X-100 and steady-state fluorimetry).

**Figure 2:**
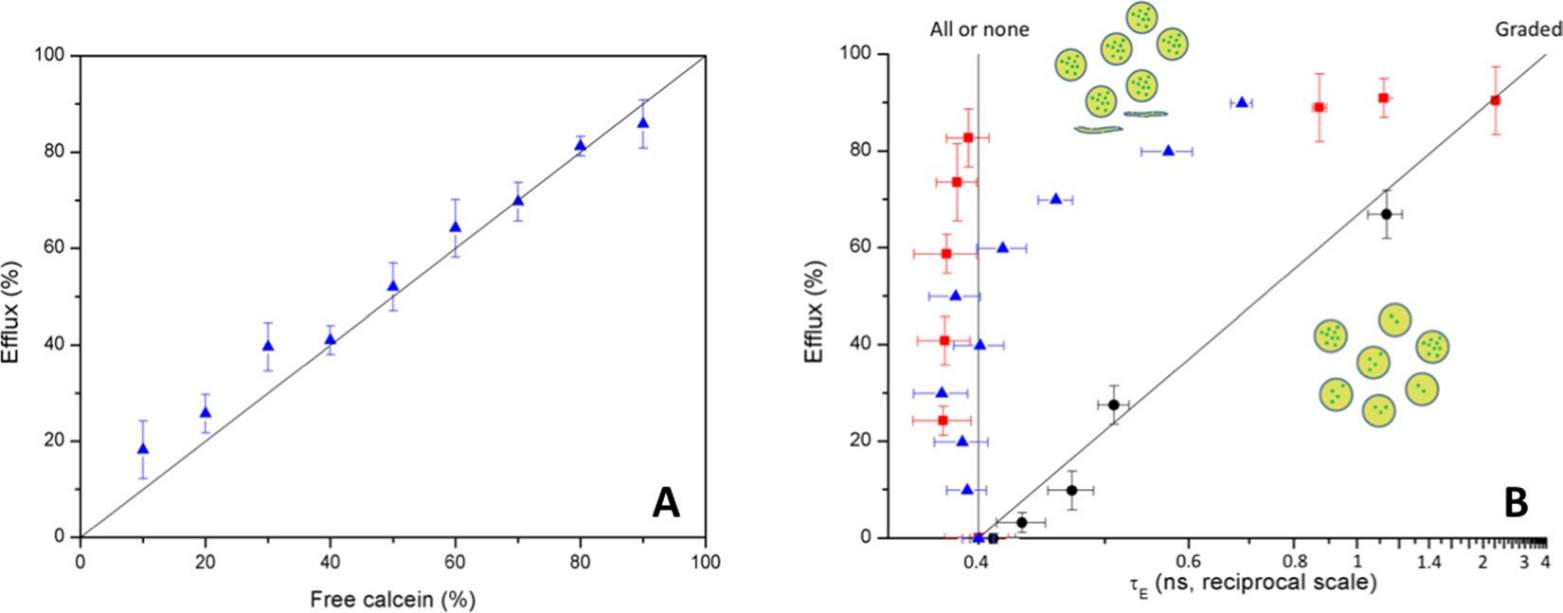
(**A**) Efflux calibration with theoretical values and (**B**) efflux as a function of lifetime for LUV + free calcein (triangles), Triton X-100 (squares), and cNP + laser (circles).

As seen in Fig. 2A, a good agreement between calculated and real values is achieved with this estimate of efflux *E*. When these values are plotted vs. the lifetime τ_E_, as shown in Fig. 2B (blue triangles), the evolution of this parameter follows the expected vertical behaviour (constant τ_E_ = 0.4 ns) for an all-or-none disruption mechanism up to 60% efflux, with some deviation for higher efflux values. The lifetime for the all-or-none mechanism remains constant at 0.4 ns up to 60% efflux because it is assumed that calcein liberated from vesicles is so diluted in the bulk of the solution that it practically does not contribute to the lifetime measurements^25^. Another approach to this disruption mechanism was performed by preparing solutions of vesicles treated with increasing concentrations of Triton X-100 between 0 and 3 μM to cause partial permeabilization of membranes. Results are presented in Fig. 2B (red squares), the behaviour does not differ significantly in trend with respect to the previous one, as expected for a solubilization process equivalent to an all-or-none mechanism.

Moreover, a graded calcein liberation from the vesicles would render a heterogeneous population carrying different inner fluorophore concentrations, thus it would be expected to presumably show a continuous increase in lifetime values (diagonal line in Fig. 2B)^29^. When vesicles were irradiated with pulsed IR laser light in the presence of different amounts of cNP, from 0 up to 80 μg/ml, lifetime measurements (plotted as a function of the calculated efflux in Fig. 2B, black circles) showed a trend clearly closer to a graded than to an all-or-none mechanism. This result supports the idea that the photoacoustic effect may cause in membranes a transient physical disruption long enough to allow some flux of liquid through it, but rapidly repaired to return to a stable integrity state. This fast healing process would be driven by thermodynamic forces, since no active cellular mechanisms are present in these model membranes.

### Calcein uptake

**Figure 3:**
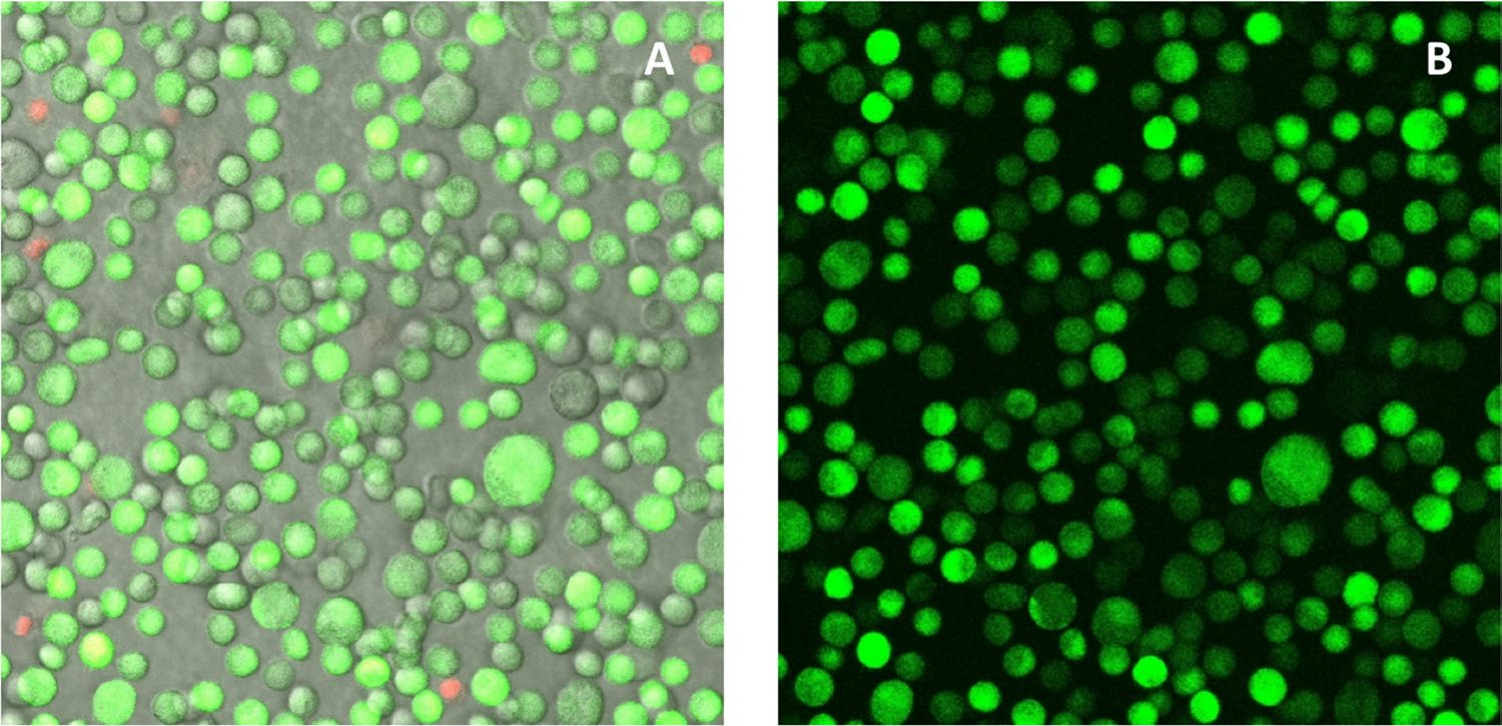
Confocal image of calcein uptake by CHO cells. (**A**) Merged image of dark field (cell contours), green (calcein) and red (propidium iodide) channels. (**B**) Same image, green channel only. Microscope software was Leica Application Suite Advances Fluorescence 2.6.3.8173, Leica Microsystems CMS, Wetzlar, Germany. https://www.leica-microsystems.com/products/microscope-software/p/leica-application-suite/.

The photoacoustic effect was tested in real cells by studying the intracellular delivery of calcein from solutions when suspended CHO cells were irradiated with pulsed IR light in the presence of cNP. Samples were eventually treated with propidium iodide to stain dead cells, and imaged in the confocal fluorescence microscope. An example is shown in Fig. 3, where 2 × 10^5^ CHO cells were irradiated with 50 mJ/cm^2^ for 60 s in the presence of 40 μg/ml cNP and 400 μM calcein. In Fig. 3A we can observe a merged imaged of dark field, green and red channels, showing, respectively, cell contours, green calcein-loaded cells, and dead cells with exposed DNA stained in red upon binding to propidium iodide. Several conclusions may be extracted from this figure. First, calcein intracellular delivery has indeed been promoted by the photoacoustic effect, since no uptake was observed in any of the controls, irradiation without cNP or no irradiation in the presence of cNP (images shown in Supplementary Materials, Fig. S2). Second, cell survival is practically complete, at least in terms of immediate cell death and integrity, since only 1–2% of cells appeared marked in red. And third, the process seems to be driven by a graded mechanism, as shown by the presence of cells with different inner concentrations of calcein, ranging from only residual or no calcein at all, to strongly bright green cells. This full gradation of colour, representative of the different calcein contents, is more easily observed in Fig. 3B, where only the green channel of the confocal micrography is displayed.

As stated in the previous section, a graded mechanism is compatible with the formation of transient openings, which would allow exchange of intra and extracellular media, driven by concentration differences at both sides of the membrane, thus resulting in net intracellular delivery or liberation. The integrity observed in these calcein-loaded cells after the whole process of irradiation, centrifugal cleaning, staining and imaging (over 1 h), corroborates the assumption that these transient membrane disruptions are fast and easily closed.

With the aim of further assessing that fluorophore uptake is controlled by diffusion, as expected from the mechanism we are proposing, different experimental conditions were assayed with respect to calcein concentration and irradiation energy, while keeping constant the other parameters. In order to quantify calcein intracellular delivery, the colour intensity per surface area of images taken in different zones of the same sample was averaged and compared with the averaged value of intensity per area extracted from zones with fully uploaded cells (representing 100% uptake). This quantification is useful for comparison purposes.

**Figure 4:**
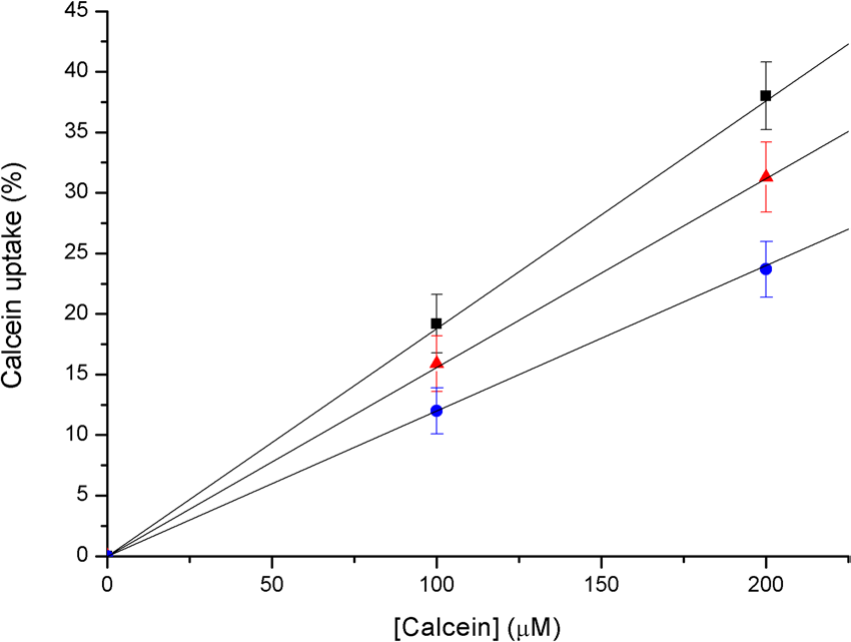
Calcein uptake vs calcein concentration in the outer medium. Laser energies: 50 (blue circles), 100 (red triangles) and 200−mJ/cm^2^ (black squares).

Results are depicted in Fig. 4, where calcein uptake, in percentage, is shown as a function of calcein concentration in the outer medium for different laser energies, namely 50, 100 and 200 mJ/cm^2^. Intracellular delivery follows a linear correlation with concentration in the three cases, as it would be expected from the first Fick’s Law of diffusion. This behaviour agrees with our assumption that intracellular delivery is governed by passive diffusion through transient membrane openings.

### Calcein release

As stated in the Introduction, multiphoton excitation in conjunction with confocal microscopy allows applying the photoacoustic effect to a sample in suspension during its optical observation/recording. To this aim, calcein-loaded and cleaned CHO cells, prepared as previously described, were mixed with fresh cNP again. Samples were then placed at the confocal microscope and, once focused, irradiated with femtosecond IR laser at a wavelength of 1000 nm for 120 s while the process was being recorded in dark field and green (515 nm) channels. In this way, the photoacoustic effect could be observed in real time. To discard the possibility of dye bleaching by any of the laser lights (pulsed IR or continuous green laser), control samples without cNP were observed and recorded in a similar way.

**Figure 5:**
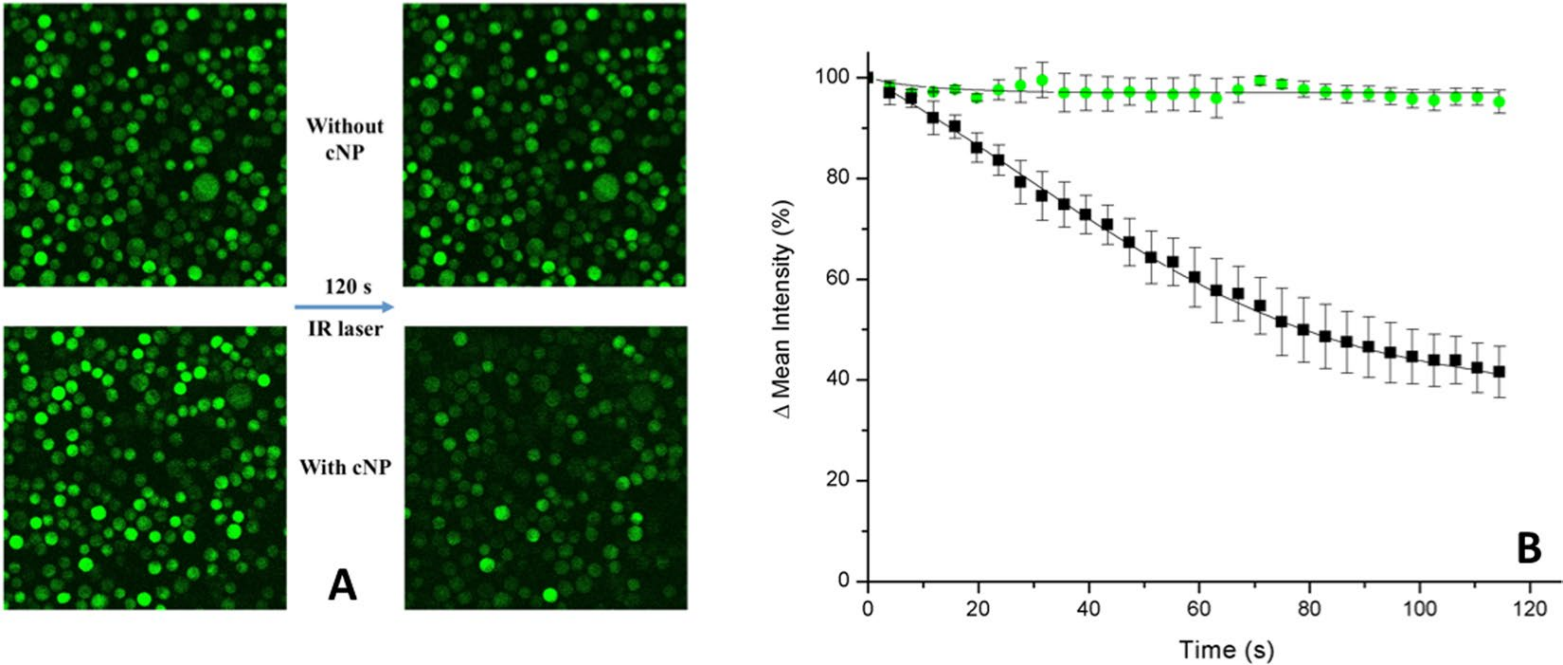
(**A**) Confocal images of cells before and after laser irradiation. (**B**) Time-course of light mean intensity for samples with (black circles) or without cNP (green squares). Average of 3 independent footages. Microscope software was Leica Application Suite Advances Fluorescence 2.6.3.8173, Leica Microsystems CMS, Wetzlar, Germany. https://www.leica-microsystems.com/products/microscope-software/p/leica-application-suite/.

Video footages obtained with this novel technique are presented in the Supplementary Materials. Figure 5A summarizes the process recorded, by showing the first and last frames after 120 s IR exposure for both the control (without cNP) and the cNP-treated samples. The bleaching that is taking place in the sample with cNP, where the photoacoustic effect is occurring, can be seen with the naked eye, while no apparent change is detected in the control. To further quantify and graphically visualize the process, mean light intensities of frames, obtained from the confocal microscope software analysis, have been plotted as a function of time for both samples, and they are presented in Fig. 5B. In the control sample no appreciable bleaching of calcein-loaded CHO cells occurs upon irradiation. However, the continuous fading of fluorescence intensity is remarkable in the sample irradiated in the presence of cNP, suggesting that calcein is being released from the CHO cells and diluted in the medium. Thus we have obtained a live recording of the photoacoustic effect producing transient openings in living cells.

### Intracellular delivery and viability

Once the occurrence of photoacoustic effect by laser-activated cNP has been established in cell membranes, it is important to know the way in which experimental parameters, namely laser energy and time of irradiation, affect cell intracellular delivery and viability,. This will help in choosing the most appropriate conditions to deliver molecules into the cells without compromising their survival. In order to improve quantification and standardization of calcein intracellular delivery and cell viability, flow cytometry was used, a convenient tool that allows the simultaneous and precise measurement of both magnitudes. Cell viability was determined not only by quantifying necrotic cells, which are stained in red with propidium iodide, but also measuring cells in early apoptosis by staining with Annexin-V labelled with the fluorescent marker FITC. We believe this is a more realistic approach to cell viability, since cells in early apoptosis should not be considered viable in the long-term.

**Figure 6:**
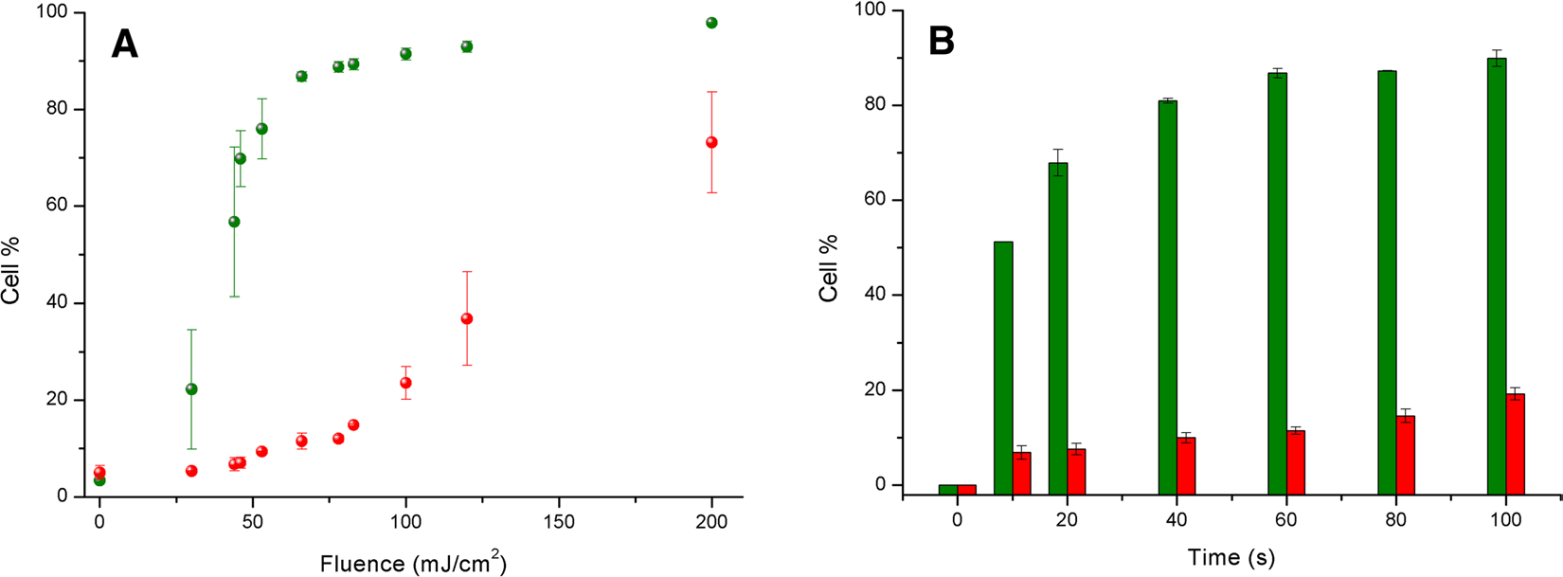
Percent calcein-loaded CHO cells (green) and viability (red) as a function of laser fluence (**A**), and as a function of time for a constant fluence of 60 mJ/cm^2^ (**B**).

In Fig. 6A we can observe the effect of laser fluence (energy per surface area) on calcein uptake and cell viability for a constant irradiation time of 60 s. As energy arises, both magnitudes increase, but at a different pace. At low fluences, intracellular delivery improves steeply, reaching a plateau around 60–70 mJ/cm^2^. However, cell viability remains at baseline values up to 50 mJ/cm^2^, then it increases gradually. This different behaviour implies that intracellular delivery may be conveniently performed, for example, at a fluence of 60 mJ/cm^2^ where uptake reaches 80% of cells but cell death or apoptosis do not exceed 10%. This behaviour supports our idea that counting dead cells alone does not seem to be an appropriate parameter to establish viability of a cell population exposed to a harsh treatment. Examples of the flow cytometry experiments represented in Fig. 6A are shown in Figs. S3 and S4 of Supplementary Materials.

The effect of laser irradiation time is presented in Fig. 6B, where samples were treated with a constant laser energy fluence of 60 mJ/cm^2^. Once again intracellular delivery increases rapidly with time at the beginning, reaching a constant value at 40 s, while cell viability decreases very slowly but steadily with time up to the maximum value assayed of 100 s (cytometry images shown in Figs. S5 and S6 of Supplementary Materials). From these results it can be concluded that, under these particular conditions, the most appropriate time of sample irradiation would be 40 s, since calcein uptake does not improve with longer exposure times but viability does decay.

### Long-term cell viability

A more reliable way of assessing the long-term viability of cells exposed to harsh conditions in suspension, not usually exploited in this kind of cellular studies, is the re-seeding on a flat surface to verify the maintenance of the properties of native, healthy cell: attaching to a surface, spreading and dividing, trying to fully cover the available area (confluence), resembling growth in a tissue. This could be a decisive experiment to test the health state of a detached and suspended cell population undergoing a stressful treatment.

**Figure 7:**
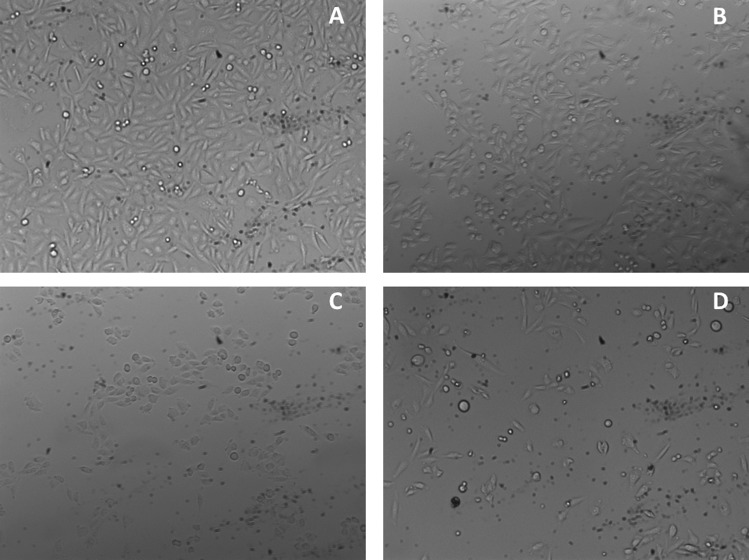
Microscope images of CHO cells after 48 h of re-seeding. Samples were exposed to different laser irradiation fluences(**A**) 0 mJ/cm^2^ (**B**) 46 mJ/cm^2^; (**C**) 66 mJ/cm^2^; (**D**) 98 mJ/cm^2^. Microscope software was Leica Application Suite Advances Fluorescence 2.6.3.8173, Leica Microsystems CMS, Wetzlar, Germany. https://www.leica-microsystems.com/products/microscope-software/p/leica-application-suite/.

Figure 7 summarizes the results obtained when CHO cells, irradiated for 60 s in the presence of 40 μg/ml cNP at different laser fluences, are re-seeded in flat flasks and grown for 48 h. There is a negative evolution of the samples with respect to the control (Fig. 7A). Figure 7B, where sample was irradiated with 46 mJ/cm^2^, shows a slight reduction in the proportion of spread cells, as well as a higher proportion of spherical cells not spread onto the surface. With stronger laser energy fluences, namely 66 and 98 mJ/cm^2^, the decrease in confluence is more marked, and these cell populations should not be considered as recovered from the laser-activated cNP treatment.

## Conclusions

The simultaneous generation of hot bubbles and shock waves arising from pulsed-laser irradiation of carbon nanoparticles (a process called “transient nanoparticle energy transduction”, TNET), in the vicinity of living cells, leads to the observation of uptake or release of cell contents, and provides a direct demonstration of the membrane perturbing effects of this photoacoustic effect. Parallel investigations using pure lipid vesicles (liposomes) support the idea that the induced membrane lesions are transient and quickly repaired, as deduced from the “graded” (*i.e*. not “all-or-none”) mechanism of dye release. Furthermore, the present study leads us to the conclusion that long-term cell survival may not be straight-forwardly determined only by assessing cell state through cytometry, even less by only focusing on counting necrotic cells. Intracellular delivery methods, which bring membranes under high stress, may provoke subtle cellular alterations that can eventually compromise cell survival. A more comprehensive investigation of the consequences of using these methods can be achieved by studying cell viability over a more extended period of time, trying to elucidate if cells retain their basic functions intact after the process.

## Materials and Methods

### Materials

Carbon nanoparticles were kindly donated by Cabot Corporation (Leuven, Belgium). Egg phosphatidylcholine (egg PC) was purchased from Lipid Products (South Nutfield, U.K.). All cell culture products: DMEM:F12 medium, fetal bovine serum (FBS), penicillin/streptomycin, GlutaMax supplement and trypsin-EDTA (0.25%) phenol red were purchased from Thermofisher (Waltham, MA, USA). Calcein, poloxamer 407, Triton X-100, Annexin V FITC apoptotic detection kit and Nuclepore Track-Etch Membranes were obtained from Sigma-Aldrich (St. Louis, MO, U.S.A.).

### Preparation and characterization of nanoparticle suspensions

Carbon Black nanoparticle (cNP) suspensions were prepared at a concentration of 0.4 mg/ml in aqueous solutions of non-ionic surfactant poloxamer 407 at 0.1% (w/v), by sonicating for 10 minutes in a bath sonicator. These highly hydrophobic carbon nanoparticles cannot be colloidally stabilized in a water dispersion without the help of a surfactant.

Size distributions of cNP suspensions were determined by dynamic light scattering (DLS) with a ZetaSizer Nano S (Malvern Instruments, Worcestershire, UK), and their morphological characteristics were assessed by transmission electron microscopy (TEM) in a JEM-2000FS/CR (Jeol Europe, Croissy-sur-Seine, France).

### Laser device

A Brilliant B pulsed Nd:YAG laser (Quantel Laser, Lannion, France) was used to irradiate samples with laser pulses of 6 ns length at a frequency of 10 Hz. Pulse energy was varied between 0 and 56 mJ/pulse by adjusting the device Q-switch delay. The wavelength of the emitted laser infrared light was 1064 nm.

The device was mounted on an optical table with a diaphragm, a convergent lens and a custom-made stand positioned to obtain a radiation spot of 6 mm in diameter. Samples (50 μl) were deposited in a Hellma Analytics black quartz cuvette (105.251-QS) with an effective area exposed to radiation of 15 mm^2^ (3 × 5 mm).

This 1064-nm laser device was used in all irradiation experiments described in this work, except in the multiphoton excitation microscopy recordings, where the built-in femtosecond IR laser was employed (at 1000 nm, maximum accessible wavelength).

### Liposome Preparation

Egg PC, dissolved in chloroform:methanol (2:1), was evaporated to dryness under a stream of nitrogen. In order to remove solvent traces the sample was kept under vacuum for 2 h, then the lipids were swollen in calcein buffer (70 mM calcein, 25 mM Hepes, pH7.4) to form multilamellar vesicles (MLV). Large unilamellar vesicles (LUV) were prepared from MLV after subjecting them to 10 freeze/thaw cycles and extrusion using Nuclepore Track-Etch Membrane filters of 100 nm pore size^30^. Non-entrapped calcein was removed from the external medium by size exclusion chromatography on a PD MidiTrap G-25 desalting column from GE-Healthcare equilibrated with a NaCl-Hepes buffer with the same pH and osmolarity of the calcein buffer (checked with an Osmomat 030 osmometer, Gonotec GmbH, Berlin, Germany).

Vesicle size was verified by DLS using the ZetaSizer Nano S device previously described. A phosphate assay was used to determine the final lipid concentration.

### Cryo-TEM sample preparation and image collection

Samples containing LUVs or LUVs and cNPs were loaded on freshly glow-discharged 300-mesh lacey-carbon coated grids (Lacey Carbon film on 300 mesh copper; LC300-Cu; Electron Microscopy Sciences). Vitrification was performed on Vitrobot Mark II (FEI Company, USA) maintained at 8 °C and at a relative humidity close to saturation (90% rH). Four microliters of sample solutions were absorbed onto the grids for 30 seconds. Then, most of the liquid in the grid was removed by blotting with absorbent standard filter paper to create an “ultra-thin liquid film” and the grid was immediately plunged into a liquid ethane bath, previously cooled with liquid nitrogen. The vitrified grids were removed from the plunger and stored under liquid nitrogen.

Imaging of cryoTEM samples was made on a JEM-2200FS/CR (JEOL Europe, Croissy-sur-Seine, France) transmission electron microscope using conditions suited for lipidic vesicles^31^. The microscope was operated at 200 kV and images were recorded under low-dose conditions, with a total dose on the order of 10–20 electrons/Å² per exposure, at defocus values ranging from −1.5 to −4.0 µm. The in-column Omega energy filter of the microscope helps to record images with improved signal-to-noise ratio (SNR) by zero-loss filtering, using an energy selecting slit width of 15 eV centered at the zero-loss peak of the energy spectra. Digital images were recorded on a 4K × 4K (15 *µm* pixels) Ultrascan4000 charge-coupled device (CCD) camera (Gatan Inc.) using DigitalMicrograph (Gatan Inc.) software, at a nominal magnification of 40,000× or 50,000× , resulting in final samplings of 2.6 Å/pixel or 2.0 Å/pixel, respectively.

### Fluorescence lifetime measurements

Fluorescence lifetime measurements were performed in a Fluoromax-3 (Horiba Jobin Yvon, Edison, USA) equipped with a 467 nm light-emitting diode pulsed at 1 Mz for excitation. Decay curves were registered using TCSPC at a wavelength of 515 nm and a bandwith of 2 nm for 180 s. The instrument response function was measured using a scattering solution of colloidal silica. Biexponential functions were used to fit the decay curves, and the fit goodness was evaluated by χ^2^ values.

### CHO cell culture

Wild type CHO-K1 (ATCC, Manassas, VA, United States) cells were grown on DMEM:F12 medium containing 10% FBS, a mixture of 100 U/ml penicillin and 100 U/ml streptomycin and 6 mM glutamine at 37 °C, under a 5% CO_2_ humidified atmosphere. CHO cells were trypsinized and collected to perform the irradiation experiments while in suspension.

### Exposition of LUV or CHO cells to the photoacoustic effect

LUV or CHO cells (10^5^ in 50 µl) were irradiated with pulsed IR light at a wavelength of 1064 nm, under different experimental conditions, in the presence of 40 μg/ml cNP unless otherwise stated. Controls with cNP but no irradiation, as well as without cNP but irradiated, were routinely performed. Measurements were done in triplicate, and samples kept in ice till they were analysed or imaged.

In the case of calcein uptake experiments by CHO cells, samples after irradiation were centrifuged at 800 rpm for 5 min and resuspended in fresh medium twice to remove excess calcein and cNP.

### Confocal and multiphoton imaging

Static images and videos were acquired on a Leica TCS SP5 II microscope (Leica Microsystems GmbH, Wetzlar, Germany). A 63x water-immersion objective (numerical aperture, NA = 1.2) was used to acquire 512 × 512 pixel images of equatorial planes (to avoid photoselection). Calcein was detected by irradiating samples with a 495 nm excitation wavelength and collected at 515 nm, while propidium iodide was recorded with 535 nm excitation light and the emission collected at 617 nm. A femtosecond pulsed titanium-sapphire (Mai-Tai Deepsee, Spectra-Physics) laser tuned at 1000 nm was used for multiphoton simultaneous imaging and IR irradiation of samples. Fluorescence emission was collected by hybrid detectors. Images were analyzed using Leica software.

### Cytometry

Fluorescence-activated cell sorting (FACS) analysis was performed using a FACS Calibur (Becton-Dickinson, Franklin Lakes, NJ) cytometer to properly quantify calcein encapsulation and cell viability after cells were subjected to the photoacoustic effect. Viability was quantified by counting cells stained with propidium iodide (necrotic state) as well as with Annexin V-FITC (early apoptosis). Since calcein and FITC display similar excitation and emission spectra, and have to be measured with the same cytometer channel (FL-1, with λ_ex_ = 488 nm and λ_em_ = 530 nm), every experiment was performed on two aliquots of the sample in parallel, one with calcein but no staining, another one without calcein but stained with both dyes, each sample in triplicate. Propidium iodide-stained cells were counted with the fluorescence channel FL-3, with λ_ex_ = 532 nm and λ_em_ = 561 nm.

Both dyes were used as indicated in the manual of the detection kit. Data analysis was performed using the Flowing Software 2 program.

### Reseeding of cells

After cell treatment by laser-activated cNP, cells were reseeded in culture flasks with the above mentioned growth medium and allowed to grow for 48 hours. After this time, transmission microscope images (Leica DMI3000), at a magnification of 20× , were taken to assess cell viability and proliferation.

## Supplementary Information

Supplementary Information.

Supplementary Information

Supplementary Information

## References

[1] Goñi FM (2014). The basic structure and dynamics of cell membranes: An update of the Singer-Nicolson model. Biochim. Biophys. Acta - Biomembr..

[2] Lodish, H. *et al*. Receptor-mediated endocytosis and the sorting of internalized proteins. In *Molecular Cell Biology 4th ed* Section 17.9 (W. H. Freeman, 2000).

[3] Galisteo-González F., Molina-Bolívar J.A., Navarro S.A., Boulaiz H., Aguilera-Garrido A., Ramírez A., Marchal J.A. (2018). Albumin-covered lipid nanocapsules exhibit enhanced uptake performance by breast-tumor cells. Colloids and Surfaces B: Biointerfaces.

[4] Panyam J, Labhasetwar V (2012). Biodegradable nanoparticles for drug and gene delivery to cells and tissue. Advanced Drug Delivery Reviews.

[5] Varkouhi AK, Marije Scholte Storm G, Haisma HJ (2011). Endosomal escape pathways for delivery of biologics. J. Control. Release.

[6] Torchilin VP (2006). Recent Approaches to Intracellular Delivery of Drugs and DNA and Organelle Targeting. Annu. Rev. Biomed. Eng..

[7] Zhang Y, Yu LC (2008). Microinjection as a tool of mechanical delivery. Curr. Opin. Biotechnol..

[8] Lentacker I, De Cock I, Deckers R, De Smedt SC, Moonen CTW (2014). Understanding ultrasound induced sonoporation: Definitions and underlying mechanisms. Adv. Drug Deliv. Rev..

[9] Gehl J (2003). Electroporation: Theory and methods, perspectives for drug delivery, gene therapy and research. Acta Physiol. Scand..

[10] Clark IB (2006). Optoinjection for efficient targeted delivery of a broad range of compounds and macromolecules into diverse cell types. J. Biomed. Opt..

[11] Chen H, Diebold G (1995). Chemical generation of acoustic waves: A giant photoacoustic effect. Science (80-.)..

[12] Chen H, McGrath T, Diebold GJ (2004). Laser Chemistry in Suspensions: New Products and Unique Reaction Conditions for the Carbon–Steam Reaction. Angew. Chemie Int. Ed. English.

[13] Sengupta A (2017). Energy Transfer Mechanisms during Molecular Delivery to Cells by Laser-Activated Carbon Nanoparticles. Biophys. J..

[14] Holguin SY, Thadhani NN, Prausnitz MR (2018). Effect of laser fluence, nanoparticle concentration and total energy input per cell on photoporation of cells. Nanomedicine Nanotechnology, Biol. Med..

[15] Sengupta A, Kelly SC, Dwivedi N, Thadhani N, Prausnitz MR (2014). Efficient intracellular delivery of molecules with high cell viability using nanosecond-pulsed laser-activated carbon nanoparticles. ACS Nano.

[16] Chakravarty P, Lane CD, Orlando TM, Prausnitz MR (2016). Parameters affecting intracellular delivery of molecules using laser-activated carbon nanoparticles. Nanomedicine Nanotechnology, Biol. Med..

[17] Chakravarty P, Qian W, El-Sayed MA, Prausnitz MR (2010). Delivery of molecules into cells using carbon nanoparticles activated by femtosecond laser pulses. Nat. Nanotechnol..

[18] Sengupta A (2015). Poloxamer surfactant preserves cell viability during photoacoustic delivery of molecules into cells. Biotechnol. Bioeng..

[19] Holguin SY, Anderson CF, Thadhani NN, Prausnitz MR (2017). Role of cytoskeletal mechanics and cell membrane fluidity in the intracellular delivery of molecules mediated by laser-activated carbon nanoparticles. Biotechnol. Bioeng..

[20] Usoltseva LO (2018). Absorption spectra of nanodiamond aqueous dispersions by optical absorption and optoacoustic spectroscopies. Photoacoustics.

[21] Duffy MJ (2018). Towards optimized naphthalocyanines as sonochromes for photoacoustic imaging *in vivo*. Photoacoustics.

[22] Chitgupi U, Lovell JF, Lovell JF (2018). Naphthalocyanines as contrast agents for photoacoustic and multimodal imaging. Biomed. Eng. Lett..

[23] Chen Q, Yu J, Kim K (2018). Review: optically-triggered phase-transition droplets for photoacoustic imaging. Biomed. Eng. Lett..

[24] Monasterio BG (2017). Coating graphene oxide with lipid bilayers greatly decreases its hemolytic properties. Langmuir.

[25] McCallion C, Burthem J, Rees-Unwin K, Golovanov A, Pluen A (2016). Graphene in therapeutics delivery: Problems, solutions and future opportunities. European Journal of Pharmaceutics and Biopharmaceutics.

[26] Francis AP, Devasena T (2018). Toxicity of carbon nanotubes: A review. Toxicology and Industrial Health.

[27] Carravilla P, Nieva JL, Goñi FM, Requejo-Isidro J, Huarte N (2015). Two-photon laurdan studies of the ternary lipid mixture DOPC:SM:Cholesterol reveal a single liquid phase at sphingomyelin:cholesterol ratios lower than 1. Langmuir.

[28] Diaspro, A. *et al*. Multi-photon excitation microscopy. *BioMedical Engineering Online* **5**, (2006). 10.1186/1475-925X-5-36 PMC1550243 16756664

[29] Patel H, Tscheka C, Heerklotz H (2009). Characterizing vesicle leakage by fluorescence lifetime measurements. Soft Matter.

[30] Nieva JL, Goñi FM, Alonso A (1989). Liposome Fusion Catalytically Induced by Phospholipase C. Biochemistry.

[31] Carreras-González A (2019). Regulation of macrophage activity by surface receptors contained within Borrelia burgdorferi -enriched phagosomal fractions. PloS Pathog..

