# Supplementary material for: Photoacoustic effect applied on model membranes and living cells: direct observation with multiphoton excitation microscopy and long-term viability analysis
Source: Sci Rep. 2020 Jan 15;10:299. doi: 10.1038/s41598-019-56799-9 (PMC6962462; doi:10.1038/s41598-019-56799-9)
Supplement: Supplementary file 1 — Supplementary Information. [file 41598_2019_56799_MOESM1_ESM.docx]

**SUPPLEMENTARY MATERIAL**

**Photoacoustic effect applied on model membranes and living cells: direct observation with multiphoton excitation microscopy and long-term viability analysis**

**Francisco Galisteo-González^1,2,3^, Bingen G. Monasterio^2,3^, David Gil^4^, Mikel Valle^4^, and Félix M. Goñi^2,3^**

^1^*Departmento de Física Aplicada, Universidad de Granada, 18570, Granada, Spain*

*^2^Instituto Biofisika (UPV/EHU, CSIC), Barrio Sarriena s/n, 48940, Leioa, Spain*

*^3^Departamento de Bioquímica y Biología Molecular, Universidad del País Vasco, Barrio Sarriena s/n, 48940, Leioa, Spain*

*^4^Structural Biology Unit, Center for Cooperative Research in Biosciences, CIC bioGUNE, Derio, Spain*


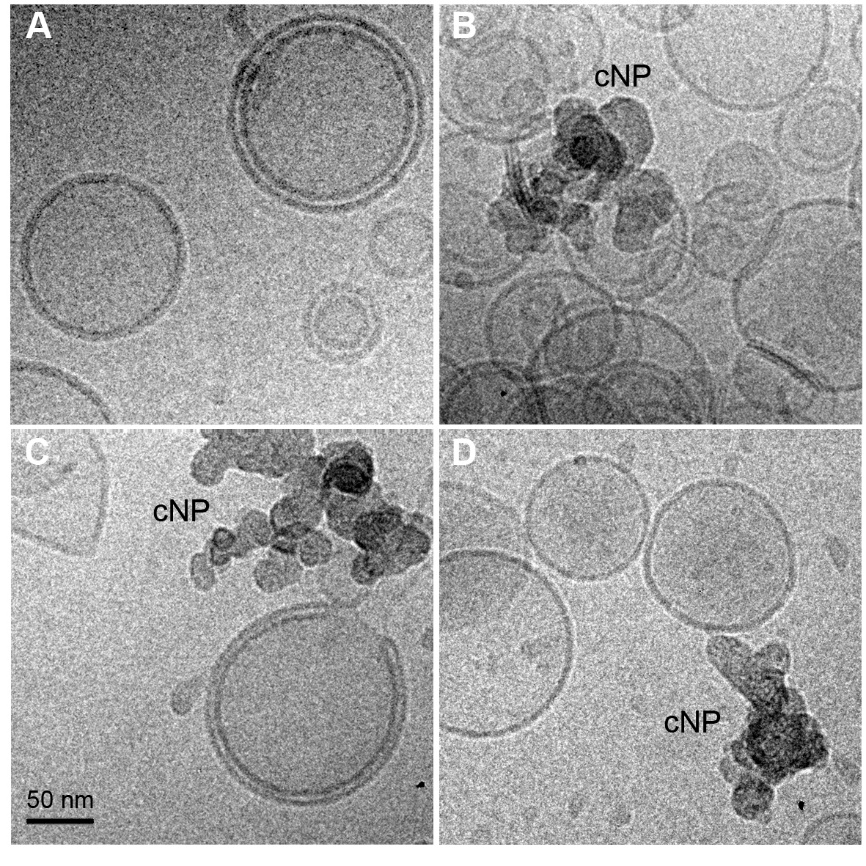


Figure S1. Image gallery for lipid vesicles in the absence (A) and in the presence (B-D) of cNPs. Images captured using DigitalMicrograph software (version 3.31.2360.0; https://www.gatan.com).


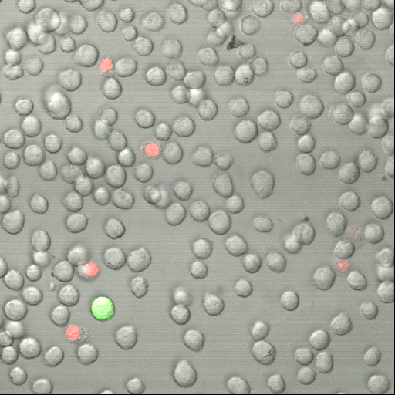

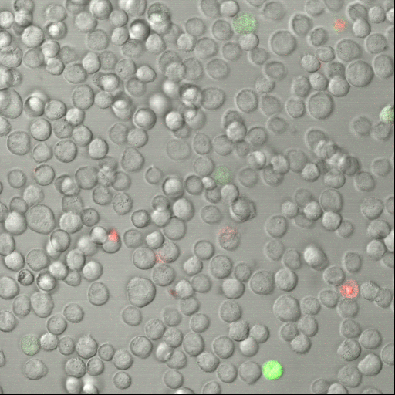


Figure S2. Control images of calcein uptake by CHO cells. (A) Irradiated with laser but without cNP. (B) In the presence of cNP but no laser irradiation.

Microscope software was Leica Application Suite Advances Fluorescence 2.6.3.8173, Leica Microsystems CMS, Wetzlar, Germany. https://www.leica-microsystems.com/products/microscope-software/p/leica-application-suite/


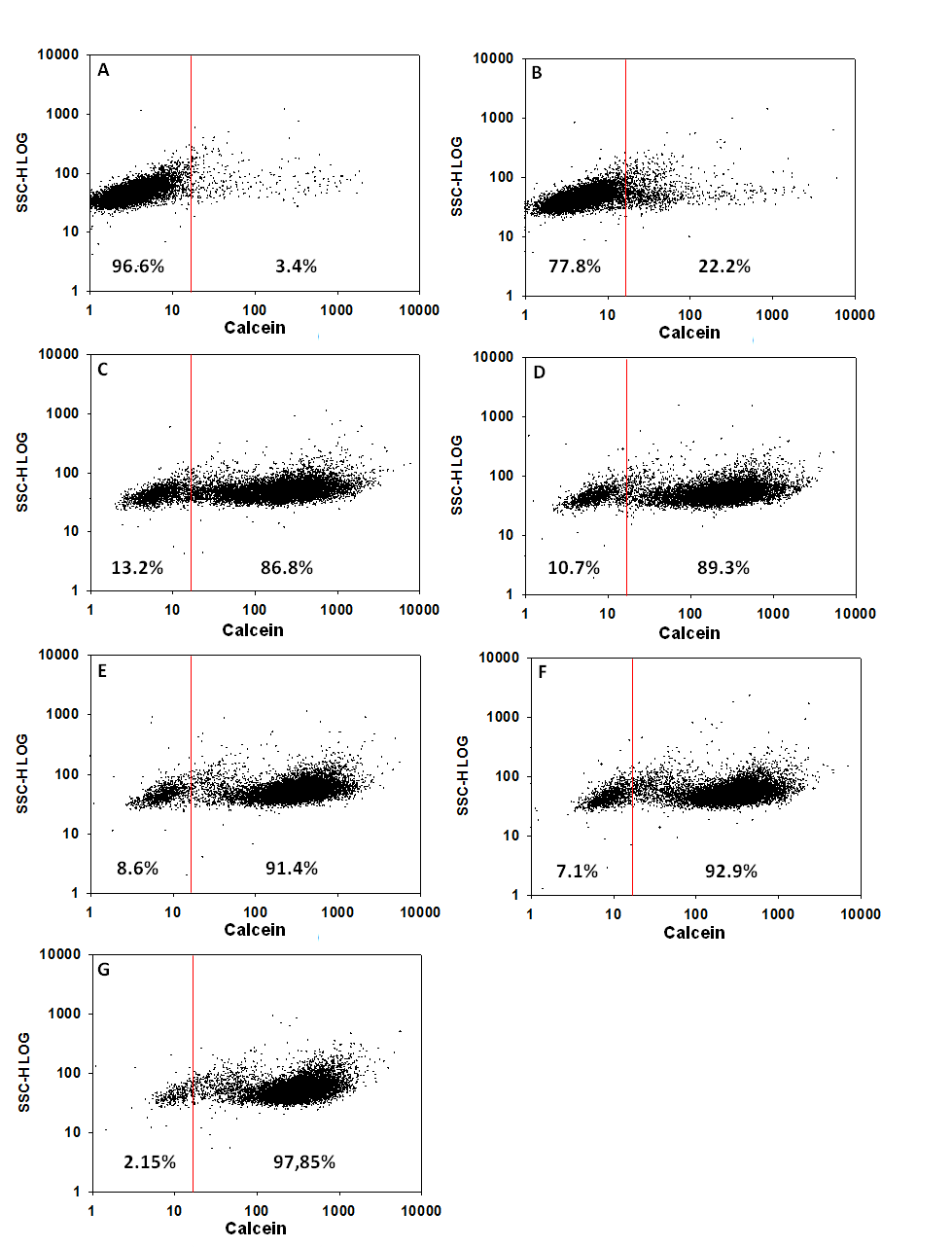


Figure S3. Calcein uptake by CHO cells. Cytometer images correspondent to Figure 6A, as a function of laser fluence (in mJ/cm^2^): A. 0; B. 30; C. 66; D. 83; E. 100; F: 120; G. 200.

FACS analysis software was Flowing software 2.5.1 Turku Centre for Biotechnology, Unversity of Turku, Finland  [http://flowingsoftware.btk.fi/](http://flowingsoftware.btk.fi/)


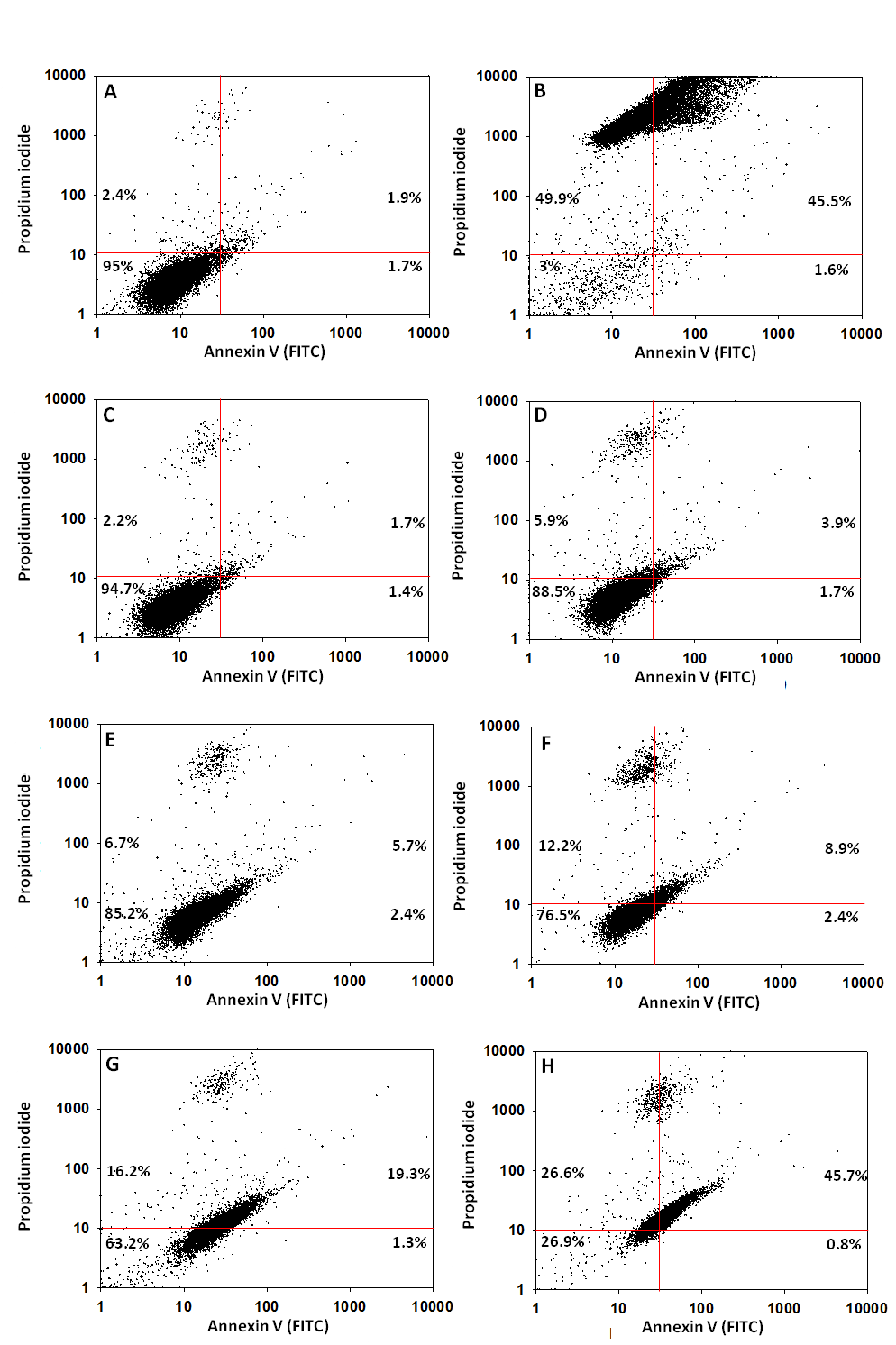


Figure S4. Viability of CHO cells. Cytometer images correspondent to Figure 6A, as a function of laser fluence (in mJ/cm^2^): A. 0; B. 100% death with ethanol; C. 30; D. 66; E. 83; F: 100; G. 120; H. 200.

FACS analysis software was Flowing software 2.5.1 Turku Centre for Biotechnology, Unversity of Turku, Finland  [http://flowingsoftware.btk.fi/](http://flowingsoftware.btk.fi/)


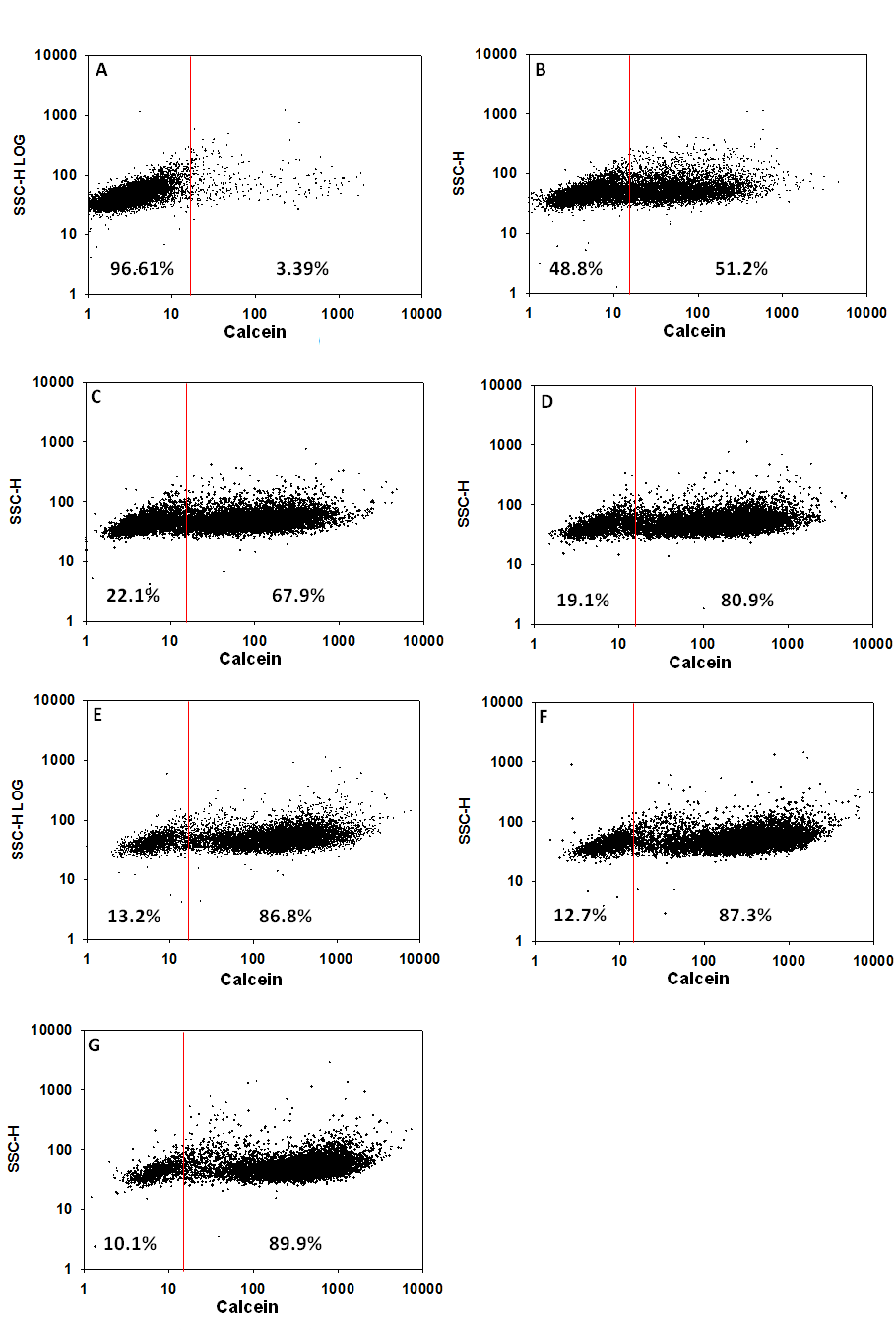


Figure S5. Calcein uptake by CHO cells. Cytometer images correspondent to Figure 6B, as a function of irradiation time (in s): A. 0; B. 10; C. 20; D. 40; E. 60; F: 80; G. 100.

FACS analysis software was Flowing software 2.5.1 Turku Centre for Biotechnology, Unversity of Turku, Finland  [http://flowingsoftware.btk.fi/](http://flowingsoftware.btk.fi/)


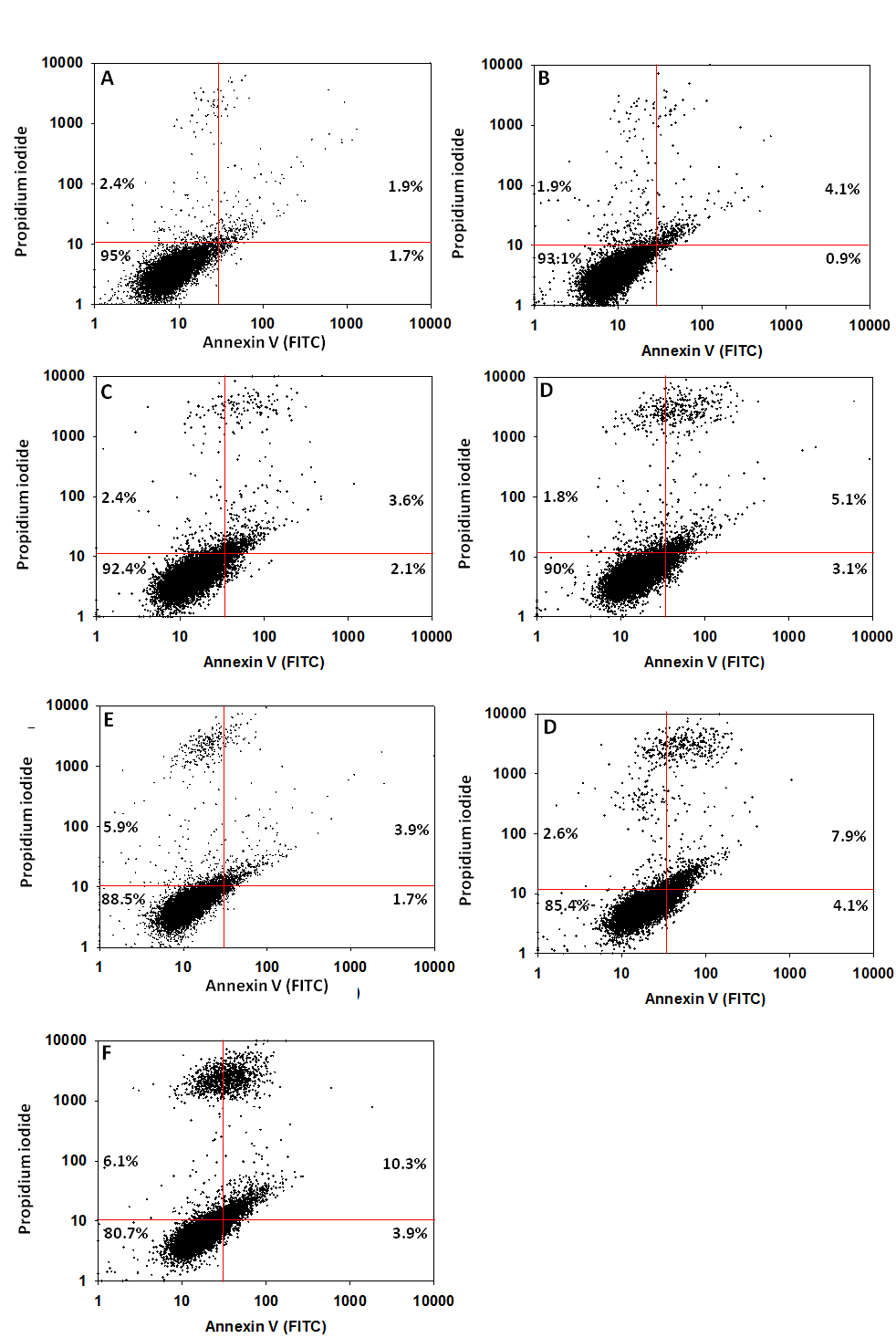


Figure S6. Viability of CHO cells. Cytometer images correspondent to Figure 6B, as a function of irradiation time (in s): A. 0; B. 10; C. 20; D. 40; E. 60; F: 80; G. 100.

FACS analysis software was Flowing software 2.5.1 Turku Centre for Biotechnology, Unversity of Turku, Finland  <http://flowingsoftware.btk.fi/>
